# DNA Extraction Protocol for Plants with High Levels of Secondary Metabolites and Polysaccharides without Using Liquid Nitrogen and Phenol

**DOI:** 10.5402/2012/205049

**Published:** 2012-11-14

**Authors:** Sunil Kumar Sahu, Muthusamy Thangaraj, Kandasamy Kathiresan

**Affiliations:** Centre of Advanced Study in Marine Biology, Faculty of Marine Sciences, Annamalai University, Tamil Nadu, Parangipettai 608 502, India

## Abstract

Mangroves and salt marsh species are known to synthesize a wide spectrum of polysaccharides and polyphenols including flavonoids and other secondary metabolites which interfere with the extraction of pure genomic DNA. Although a plethora of plant DNA isolation protocols exist, extracting DNA from mangroves and salt marsh species is a challenging task. This study describes a rapid and reliable cetyl trimethylammonium bromide (CTAB) protocol suited specifically for extracting DNA from plants which are rich in polysaccharides and secondary metabolites, and the protocol also excludes the use of expensive liquid nitrogen and toxic phenols. Purity of extracted DNA was excellent as evident by A260/A280 ratio ranging from 1.78 to 1.84 and A260/A230 ratio was >2, which also suggested that the preparations were sufficiently free of proteins and polyphenolics/polysaccharide compounds. DNA concentration ranged from 8.8 to 9.9 *μ*g *μ*L^−1^. The extracted DNA was amenable to RAPD, restriction digestion, and PCR amplification of plant barcode genes (matK and rbcl). The optimized method is suitable for both dry and fresh leaves. The success of this method in obtaining high-quality genomic DNA demonstrated the broad applicability of this method.

## 1. Introduction

The isolation of pure, intact, and high-quality DNA is very crucial for any molecular studies [1]. However, DNA isolation from plants is usually compromised by excessive contamination by secondary metabolites. The DNA isolation methods need to be adjusted to each plant species and even to each plant tissue because of the presence of these metabolites, unlike animals and microbes [2]. The search for a more efficient means of extracting DNA of both higher quality and yield has led to the development of several protocols for isolating DNA from plants containing high levels of secondary metabolites [3–7]. The mangroves and salt marsh are specially adapted to harsh environment such as marshy anoxic anaerobic soil and fluctuating salinity of the water bodies [8]. To avoid these stress conditions mangroves and salt marsh plants synthesize high amounts of polysaccharides, polyphenols, and other secondary metabolites such as alkaloids and flavanoids which impede DNA extraction [9, 10]. 

Many factors can cause shearing of DNA during extraction. Degradation of DNA due to endonucleases is one such problem encountered in the isolation and purification of high molecular weight DNA from plant, which directly or indirectly interfere with the enzymatic reactions [11]. Polysaccharides may be particularly problematic when present in DNA samples, as their presence may also inhibit enzymatic activity. Presence of polysaccharides has been shown to inhibit *Taq* polymerase activity [12] and restriction enzyme activity [13]. The presence of polysaccharides in the DNA sample is characterized by formation of a highly viscous solution [14]. The oxidized form of polyphenols covalently binds to DNA giving a brown colour and reduces maintenance time, making it useless for molecular studies [15].

Apart from traditional extraction approaches, several commercial kits are also accessible to extract genomic DNA from plants with sufficient quality [16]. For extracting genomic DNA an initial mechanical grinding of the leaf sample is carried out in the presence of liquid nitrogen, where the ultimate aim is to access the nuclear material without degradation. Numerous DNA isolation protocols use phenol to separate cellular molecules and debris from the DNA which is toxic, hazardous, expensive, and require special containment facilities to maximize personnel safety and minimize environmental concerns. However, the convenience provided by the above-mentioned methods may be cost prohibitive when considering experiments with limited financial resources.

Several researchers have attempted to eliminate the use of hazardous chemicals, expensive kits, equipment, and labour-intensive steps for high throughput DNA extraction. However, these methods do have demerits such as limited shelf life, low purity, low recovery, and poor amplification [17, 18]. Mostly the DNA extraction protocols recommend fresh leaf samples for genomic DNA isolation, but it seems impractical when the samples are collected from remote and rare locations. These situations necessitate the development of the protocols for isolating DNA from dried leaf samples. The objective of this study was to develop a simple method to isolate DNA in an open laboratory environment, a method that eliminates the need to use liquid nitrogen and toxic phenol. The resulting optimized CTAB (Cetyl trimethylammonium bromide) protocol enables the isolation of high quality genomic DNA amenable to RAPD (Random amplified Polymorphic DNA), restriction digestion, and amplification of plant barcode genes (matK and rbcl) with reduced cost and health concerns.

## 2. Materials and Method

### 2.1. Plant Samples for DNA Isolation

Young, tender, and unbruised leaves of mangroves (*Rhizophora mucronata, Rhizophora apiculata, Aegiceras corniculatum, Lumnitzera racemosa, Lumnitzera littorea, Bruguiera gymnorrhiza, Bruguiera cylindrica, Scyphiphora hydrophyllacea, Avicennia marina, Avicennia officinalis,* and *Xylocarpus mekongensis*) and salt marsh (*Suaeda maritima* and *Sesuvium portulacastrum*) were collected from Pichavaram Mangrove Forest, Tamil Nadu, India and stored in −80°C until use. The leaves were preferred for DNA extraction due to their continued availability whole year round. A minimum of ten accessions were taken for each genus. Dried leaves of *Rhizophora* and *Avicennia* sp. were also taken to scrutinize the applicability of the optimized extraction protocol.

### 2.2. Extraction Methods

Plant genomic DNA extraction Kit (GeNei), CTAB DNA extraction method by Porebski et al. [4], J. J. Doyle and J. L. Doyle [19], and Saghai-Maroof et al. [20] were employed for extracting DNA from the study plants. Among all the tested protocols, Saghai-Mahroof method yielded convincing results. Therefore, this method was taken and optimized for DNA extraction by varying the concentration of Tris-HCl, NaCl (Sodium Chloride), *β*-mercaptoethanol, and PVP (PolyVinyl Pyrrolidone).

### 2.3. Standardized Extraction Method

(i) Preheat suspension buffer (pH 8) containing 50 mM EDTA, 120 mM Tris-HCl, 1 M NaCl, 0.5 M sucrose, 2% Triton-X 100, and 0.2%  *β*-mercaptoethanol (to be freshly added just before use) in water bath at 60°C.
(ii) Grind −80°C stored leaves (1 g) to fine powder in ice cold condition in the presence of 250 mg PVP (PolyVinyl Pyrrolidone, Mr 10,000) by using pre chilled mortar and pestle (−40°C/−80°C).

*Note:* To avoid usage of liquid nitrogen, this method is successfully employed. If −80°C is not available, −40°C/−20°C can also be used. Lower the temperature and lower will be the chances of DNA degradation (nuclease activity). Appearance of brown colour indicates DNA degradation.

(iii) Transfer the content in 2 mL microcentrifuge tubes and suspend in two volumes of suspension buffer.
(iv) Invert and mix gently and incubate at 60°C for 40 min.
(v) Centrifuge the suspension at 10,000 rpm for 15 min at room temperature.
(vi) Add 1.5 mL of extraction buffer containing 20 mM EDTA, 100 mM Tris-HCl, 1.5 M NaCl, 2% CTAB, 1%  *β*-mercaptoethanol and incubate at 60°C for 30 min.
(vii) Centrifuge at 12,000 rpm for 15 min at room temperature.
(viii) Carefully transfer the aqueous phase into a new tube.

*Note:* Use wide-bore tips for transferring the aqueous phase to avoid mechanical damage to DNA.

(ix) Add double volume of Chloroform: Isoamyl alcohol (24 : 1), and invert gently 15 to 20 times and centrifuge at 12,500 rpm for 15 min.

*Note:* If the aqueous layer appears translucent, repeat the step until the solution is transparent.

(x) Add double volume of chilled isopropanol and keep at −20°C for one hour to precipitate the DNA.

*Note:* The longer the chilled incubation, the more the precipitation.

(xi) Centrifuge at 12,000 rpm for 15 min and discard the supernatant.
(xii) To the pellet, add 70% chilled ethanol and spool out the pellet carefully and centrifuge again at 12,000 rpm for 15 min.
(xiii) Discard the supernatant and vacuum dry or air dry the pellet at room temperature.

*Note:* Make sure that there is no residual ethanol, this is very critical especially if the DNA is to be used directly for PCR. Overdrying should also be avoided as it makes the pellet difficult to resuspend.

(xiv) Add 100 *μ*L of high salt TE buffer (0.5 M NaCl, 10 mM Tris-HCl, 1 mM EDTA (pH 8).
(xv) Add 3 *μ*L RNase (10 mg/mL) and keep at 37°C for 30 min followed by chloroform: isoamyl alcohol extraction and ethanol precipitation in the presence of 3 M sodium acetate (pH 5.2).
(xvi) Spool out the DNA, wash in 70% ethanol, air or vacuum dry.
(xvii) Add 30 to 50 *μ*L (depending upon the pellet) of TE buffer (10 mM Tris-HCl, 1 mM EDTA, pH 8) to dissolve the precipitate.

*Note:* Chelator present in TE can affect PCR and restriction digests. DNA in TE should be suitably diluted before use in such reactions.

(xviii) Store at −20°C/−40°C till further use.

### 2.4. Qualitative and Quantitative Analysis of Extracted DNA

The DNA yield was measured by using a UV-Visible spectrophotometer (Perkin Elmer) at 260 nm. DNA purity was determined by calculating the absorbance ratio A260/280. Polysaccharide contamination was assessed by calculating the absorbance ratio A260/230 [21]. For quality and yield assessments, electrophoresis was done of all DNA samples in 0.8% agarose gel, stained with Ethidium Bromide and bands were observed in gel documentation system (Alpha Innotech).

### 2.5. Random Amplified Polymorphic DNA (RAPD) Study

The PCR amplification reaction was carried out with five random decamer primers (*Rpl1 *to *Rpl5*) obtained from GeNei (Bangalore) in a 25 *μ*L reaction volume containing 10 mM Tris-HCl, pH 8.3, 2.5 mM MgCl_2_, 1 mM dNTP mix, 0.2 *μ*M of each primer, 1 U of *Taq* DNA polymerase, and 15 to 40 ng of template DNA. RAPD-PCR was performed in a thermalcycler (Tech Gene) for 40 cycles consisting of denaturation at 94°C for 30 sec, annealing at 45°C for 30 sec, and extension at 72°C for 60 sec. The final extension was carried out at the same temperature for 5 min. The amplified product was checked in 1.5% agarose gel electrophoresis and bands were observed in gel documentation system (Alpha Innotech).

### 2.6. Restriction Fragment Length Polymorphism (RFLP) Study

The extracted DNA was subjected to RFLP study [22]. Briefly, the reaction mixture was prepared by adding 10 *μ*L of extracted DNA, 15 *μ*L of 2X assay buffer, 10 *μ*L of BSA, and 3 *μ*L of restriction enzyme (*Bam *hI,* ECO*RI and* Pst*I). The vials were incubated at 37°C for 1 hr for complete digestion. The restriction enzyme digested products were visualized through silver staining of the polyacrylamide gel. The gels were fixed in 50 mL of fixing solution (diluted five times with 30.4 mL double-distilled water and 9.6 mL ethanol) for 30 min and silver-impregnated (with 1X staining solution) for another 30 min. This was followed by washing the gels in double-distilled water for 1 to 2 min. After removing the staining solution the gels were then kept in the developing solution in dark for 10 min. When the bands were dark enough, the developing solution was poured out and the stopping and preserving solution was immediately added. 

### 2.7. matK and rbcl Gene Amplification

PCR amplification of matK (trnK-F: gggttgctaactcaatggtagag; trnK-R: tgggttgcccggggccgaac) and rbcl (rbcl-F: actgtagtaggtaaacttgaaggtgaacg; rbcl-R: gaaccttcctcaaaaaggtctaaggggta) were carried out in 25 *μ*L reaction containing 1.0 U *Taq* DNA polymerase, 1 mM dNTPs-Mix, 1X*Taq* buffer, 2.5 mM MgCl_2_, 20 mM of each amplification primer, and 10–50 ng of template DNA in a one-step touchdown PCR-program (1 cycle at  90 sec at 96°C, 60 sec at 50°C, 120 sec at 68°C, 35 cycles at 30 sec at 95°C, 60 sec at 48°C, 120 sec at 68°C, subsequent final elongation of 20 min at 68°C) [23]. Annealing temperature (*T*_*a*_) ranged from 47 to 50°C with respect to different plant species. The amplified products were separated by electrophoresis in 1.5% agarose gel buffered with 1X TAE. Gels were stained with ethidium bromide, and bands were observed in gel documentation system (Alpha Innotech).

## 3. Results and Discussion

**Figure 1:**
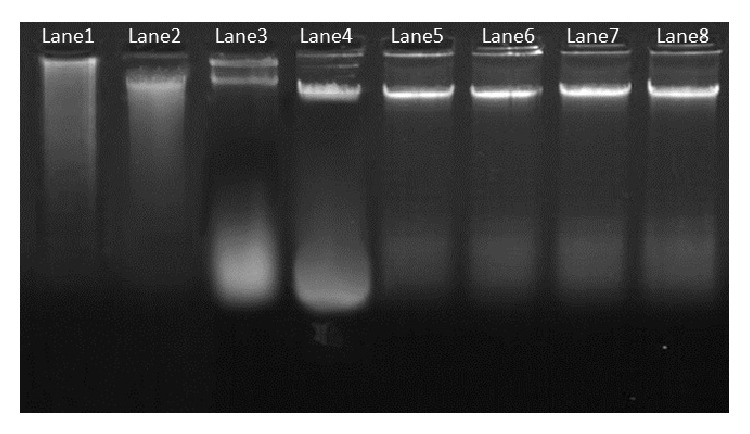
Genomic DNA isolated from plant leaves resolved under 0.8% agarose gel. Lane1 shows the DNA isolated by using Plant genomic DNA extraction Kit (GeNei); Lane2, Lane3, and Lane4 shows the isolated DNA by the CTAB method described by J. J. Doyle and J. L. Doyle [19]; Porebski et al. [4]; and Saghai-Maroof et al. [20] respectively. Lane5 to Lane8 represents the isolated DNA by the present optimized extraction method (Lane1 to Lane4 represents *A. marina*; Lane5 represents the isolated DNA from salt marsh (*Suaeda maritima*); Lane6 and Lane7 illustrate the isolated genomic DNA from the fresh leaves of *A. marina*, whereas Lane8 represents the DNA isolated from dried leaves of *A. marina*).

Plant genomic DNA extraction Kit (GeNei) did not show promising results for mangroves and salt marsh species as evident by the presence of sticky polysaccharides in the pellet and sheared band in the agarose gel. We encountered many difficulties from the very first step of cell lysis to DNA separation in the supernatant and subsequent reactions when the CTAB DNA extraction method of Porebski et al. [4] and J. J. Doyle and J. L. Doyle [19] was followed. Highly viscous and sticky pellets were difficult to handle and the brownish pellet, indicated contamination by phenolic compounds [24]. The amount of DNA obtained with these protocols was very low, and the quality was also poor for most of the samples. A260/A280 ratio was less than 1.6, that is, below the optimal limit of 1.8 [25] making the DNA nonamenable for molecular studies. But, interestingly the CTAB method described by Saghai-Maroof et al. [20] gave better DNA yield in terms of quality and quantity from the study plants. Hence, this method was considered for the purpose of standardization at varying concentration of Tris-HCL, *β*-mercaptoethanol, NaCl, and PVP (Figure 1). 

The success of the optimized extraction method in obtaining high-quality genomic DNA from all the tested mangrove and salt marsh species demonstrated the broad applicability. DNA concentration of the extraction method ranged from 8.8 to 9.9 *μ*g *μ*L^−1^. The use of prechilled mortar and pestle and −40°C/−80°C stored leaf sample successfully substituted the use of costly liquid nitrogen. The final DNA pellets were white with no visible discoloration. It has been reported that high level of *β*-mercaptoethanol successfully removes the polyphenols [26]. Therefore, high concentration of *β*-mercaptoethanol was used which made the protocol good for extraction of high-quality DNA. The addition of NaCl at concentrations higher than 0.5 M, along with CTAB, is known to remove polysaccharides during DNA extraction [24, 27]. The concentration of NaCl varied with plant species in a range between 0.7 M [28] to 6 M [29]. In the present study, higher level of NaCl (1.5 M) in the extraction buffer further improved the quality of the extracted DNA. The high quality of obtained DNA could also be attributed to the use of a higher concentration of PVP (2.5%) with lower molecular weight (10,000) rather than 40,000. A number of workers [30, 31] have recommended the use of PVP with molecular weight of 10,000 at 2% (w/v) to address the problem of phenolics. PVP with low molecular weight has less tendency of precipitating with the nucleic acids as compared to PVP with high molecular weight thus yielding sufficient amount of polyphenol-free DNA [32].

**Figure 2:**
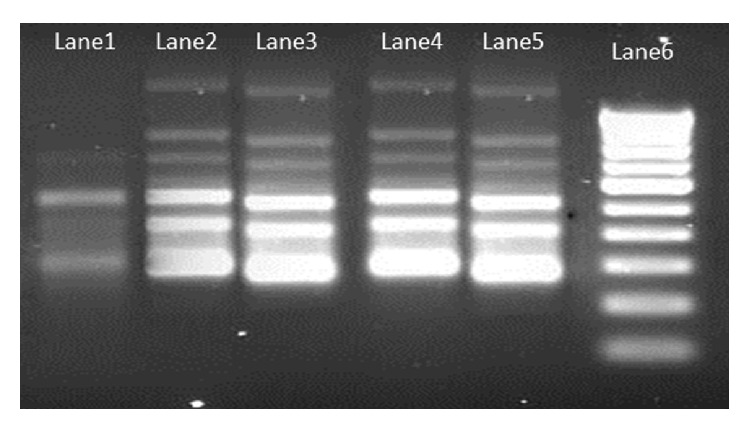
RAPD pattern of mangroves and salt marsh genotypes (Lane1: Salt marsh (*S. maritima*); Lane2 to Lane5: Mangroves (*A. marina*); Lane6: 100 bp ladder).

**Figure 3:**
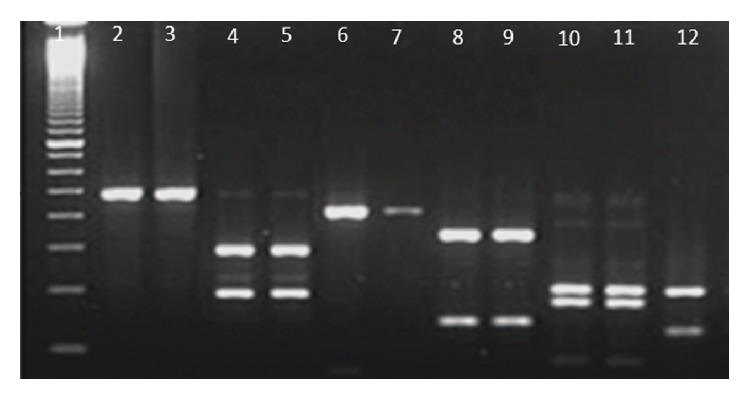
Restriction patterns of the extracted DNA cleaved by *Bam*HI, *Eco*RI and *Pst*I. Lane1: 100 bp ladder; Lane2 and Lane3: *Bam*HI cleaved mangrove DNA; Lane4 and Lane5: *Eco*RI cleaved mangrove DNA; Lane6 and Lane7: *Pst*I cleaved mangrove DNA; Lane8 to Lane12 represents the restriction pattern produced by salt marsh species DNA by *Bam*HI, *Eco*RI and *Pst*I repectively.

**Figure 4:**
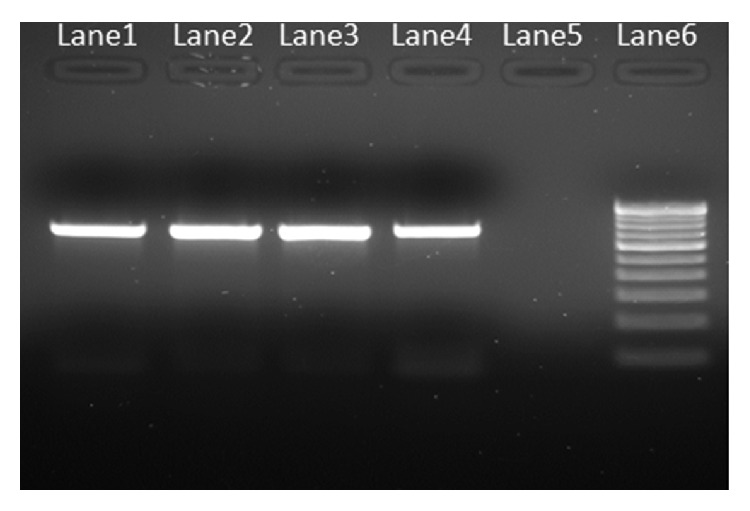
Agarose gel (1.5%) showing the PCR amplification of representative DNA samples. (Lane1 and Lane2: mangroves matK and rbcl gene respectively; Lane3 and Lane4: salt marsh's matK and rbcl gene respectively; Lane5: Negative control; Lane6: 2 Kb DNA ladder).

Purity of extracted DNA was excellent as evident by A260/A280 ratio ranging from 1.78 to 1.84 and A260/A230 ratio was >2, suggesting that the preparations were sufficiently free of proteins and polyphenolics/polysaccharide compounds [20]. Clear banding patterns were observed in the RAPD study (Figure 2). The extracted DNA was also amenable for RFLP study and amplification of plant barcode genes as evident in Figures 3 and 4, respectively. The successfully amplified barcode genes sequences were sequenced and submitted to Genbank (Accession numbers: JQ433951, JQ421082, JQ511854, and JQ421083)

 Fresh and young leaf materials are the first choice to obtain good-quality DNA. However, mature leaves contain higher quantities of polyphenols and polysaccharides [4], which makes it very difficult to isolate DNA of good quality. However, availability of young leaves for the molecular studies is quite challenging for some species. Overcoming this issue using the present optimized protocol yielded better quality DNA even from the dried-leaf samples. No DNA fragmentation due to shearing of DNA during extraction procedure was seen in any of samples and results were reproducible. This absence of smears further substantiates the high purity of extracted DNA. It has been reported previously that shearing of DNA during extraction can directly or indirectly interfere with the enzymatic reactions [11] during different molecular studies, that is, PCR, RFLP, and RAPD and so forth. No inhibition of *Taq* DNA polymerase activity was observed. Repeated chloroform: isoamylalcohol treatment ensured removal of chlorophyll, pigments, and dyes. This protocol could also be useful in other plant species with high polysaccharide and secondary metabolites during the DNA extraction process.

## 4. Conclusion

Here we have described a simple, safe, reliable, and cost-efficient CTAB DNA extraction method that provides high-quality DNA from recalcitrant mangroves and salt marsh plants containing elevated concentrations of polysaccharide and polyphenolic compounds. This method eliminates the need to use expensive liquid nitrogen and environmentally hazardous phenol to obtain high-quality genomic DNA. The proposed method enables the extraction of DNA even from dried leaves of mangroves and saltmarsh. The resulting optimized CTAB protocol enables the isolation of high quality genomic DNA amenable to RAPD, RFLP, and amplification of plant barcode genes (matK and rbcl). Therefore this method is recommended even in low-technology laboratories for high-throughput sample preparation suitable for various molecular analytical techniques.
